# Validation of the Polish version of the DREEM questionnaire – a confirmatory factor analysis

**DOI:** 10.1186/s12909-023-04539-z

**Published:** 2023-08-15

**Authors:** Dorota Wójcik, Leszek Szalewski, Adam Bęben, Iwona Ordyniec-Kwaśnica, Sue Roff

**Affiliations:** 1 Department of Dental Prosthetics, Medical University of Lublin, Lublin, Poland; 2 Digital Dentistry Lab, Department of Dental and Maxillofacial Radiodiagnostics, Medical University of Lublin, Lublin, Poland; 3 Department of Dental Prosthetics, Medical University of Gdańsk, Gdańsk, Poland; 4 Centre for Medical Education, University of Dundee, Dundee, Scotland UK

**Keywords:** Dental students, DREEM questionnaire, Learning environment, Medical education

## Abstract

**Aim:**

The aim of our study was to translate and adapt the Dundee Ready Education Environment Measure (DREEM) questionnaire developed by Roff et al. to the cultural conditions in Poland and also to validate it. Studying the learning environment is beneficial because it can identify students’ perceptions of their environment and support the staff in reflecting on, planning for and combining proper teaching approaches to improve it.

**Methods:**

The DREEM questionnaire was completed by students of all years (first–fifth) in the faculties of dental medicine at the Medical University of Lublin and the Medical University of Gdańsk. The total surveyed population consisted of 650 students. Validity was separated into four phases: (1) translation validity, (2) confirmatory factor analysis, (3) concurrent validity and (4) criterion-related validity.

**Results:**

Our study confirmed the original structure of the DREEM tool (GFI = 0.955, AGFI = 0.951, NFI = 0.931, TLI = 0.962, CFI = 0.964, RNI = 0.964, IFI = 0.964, RFI = 0.928, PNFI = 0.885, SRMR = 0.062, RMSEA = 0.043, 90% CI = 0.041–0.046) and obtained very good reliability rates, with Cronbach’s alpha > 0.7 for all scales. Only Subscale V achieved a lower Cronbach’s alpha of > 0.5. The study was conducted using the test–retest method, which is why the intraclass correlation coefficients for reliability were also calculated; individual items showed both medium and good correspondence.

**Conclusions:**

Our study provided good evidence for the reliability and validity of the Polish version of the DREEM. In conclusion, the Polish-language version of the DREEM questionnaire is a reliable and valid instrument for analysing the learning environment for dental students and its factor structure is supported by the data.

## Background

Today, a positive learning environment is seen as an important element of a student’s education due to its higher education efficiency, which enables students to achieve better learning outcomes and greater satisfaction [1, 2].

The Dundee Ready Education Environment Measure (DREEM) questionnaire was developed by Roff et al. in 1997 [3]. The aim was to develop and validate a universal diagnostic inventory for assessing the whole or parts of the educational environment of health professions / medical schools and to enable evaluation of their responses to the challenges of changing mandates and missions. The DREEM questionnaire was created using a standard methodology based on grounded theory, in cooperation with almost 100 medical professionals from all over the world, and validated by over 1000 students from Scotland, Argentina, Bangladesh, and Ethiopia. The survey consists of five parts: (I) students’ perception of teaching, (II) students’ perception of teachers, (III) students’ academic self-perception, (IV) students’ perception of the atmosphere and (V) students’ social self-perception. It contains 50 statements evaluated by the respondents on a five-point Likert scale [3]. As of 2005 (2), the validated survey was available in Spanish [4, 5], Persian [6], Chinese [7], German [8], Greek [9], Indonesian [10], and Korean [11]. In many countries, the English version of the questionnaire was used [12–14].

The Johns Hopkins Learning Environment Scale developed by Robert B. Shochet, Jorie M. Colbert and Scott M. Wright of the John Hopkins University School of Medicine consists of 28 items that are used to evaluate perception of the academic environment [15]. Development of the JHLES survey began in 2012 with the use of standard methodology. Its items are graded using a Likert scale. The objective of the JHLES is to assess students’ perception of the institutional curriculum, atmosphere and opportunities, the relations with peers and university staff, and the level of involvement in the academic community. The JHLES was translated, adapted, and used in several countries, such as Brazil [16], China [17], and Malaysia [18, 19]. To date, the tool has not been translated into Polish or validated for Polish conditions.

The aim of our study was to translate and adapt the DREEM questionnaire to cultural conditions in Poland and to validate it. Careful examination of the educational environment is essential for ensuring improved quality of the curriculum. The most widely used and readily available tool for analysing the educational environment is the assessment of how such environment is perceived by students [20,21]. The Polish educational environment has not seen such assessment conducted on a large scale. Adopting adequate research tools is necessary for carrying out an analysis of the educational environment and our study will allow for both methods to be used in examining the educational environment of Polish medical schools in the future. Studying the learning environment is beneficial because it can identify students’ perceptions of their environment and support the staff in reflecting on, planning for and combining proper teaching approaches to improve it.

The DREEM is an instrument that is commonly used to evaluate the learning environment of the medical sciences and other health sciences in various academic settings. The results are used to compare different institutions that offer health courses. DREEM can be used for assessing students' opinions on medical education. Moreover, it has proven to be a successful instrument for identifying curricular imperfections and evaluating the implementation of curricular changes [23, 24]. It has also served as a tool for identifying discrepancies between students' expectations and educational experiences [25]. Receiving feedback from students through the DREEM research system allows changes to be made in the learning environment of medical universities [18]. This tool has been successfully implemented in undergraduate courses by professionals and also generally in healthcare fields, including medicine, dentistry, nursing, midwifery, anesthesiology, medical emergencies, paramedical, including medicine, dentistry, nursing, midwifery, anaesthesiology, medical emergencies, paramedical sciences and chiropractic learning environments [22]. To the best of the authors' knowledge, the dentistry population and other medical students examined in this study have not been involved in any other validated form of assessment of the educational environment. It was therefore decided to include all the dentistry students from the two selected universities in this study.

## Material and methods

The aim of our study was to validate the questionnaire, translate it into Polish, and adapt it to the cultural differences. There are no universal guidelines for intercultural adaptation, therefore the methodology adapted from previous studies was implemented.

The original DREEM questionnaire is shown in Table 1.

**Table 1:** The original Dundee Ready Education Environment Measure (DREEM) – items grouped by subscale

Scale	Item	Question
Subscale I: Students’ Perception of Learning	1	I am encouraged to participate during teaching sessions
	7	The teaching is often stimulating
	13	The teaching is student-centered
	16	The teaching helps to develop my competence
	20	The teaching is well focused
	22	The teaching helps to develop my confidence
	24	The teaching time is put to good use
	25^a^	The teaching over-emphasises factual learning
	38	I am clear about the learning objectives of the course
	44	The teaching encourages me to be an active learner
	47	Long-term learning is emphasised over short-term learning
	48^a^	The teaching is too teacher-centered
Subscale II: Students’ Perception of Teachers	2	The teachers are knowledgeable
	6	The teachers adopt a patient-centred approach to consulting
	8^a^	The teachers ridicule the students
	9^a^	The teachers are authoritarian
	18	The teachers have good communication skills with patients
	29	The teachers are good at providing feedback to students
	32	The teachers provide constructive criticism here
	37	The teachers give clear examples
	39^a^	The teachers get angry in teaching
	40	The teachers are well prepared for their teaching sessions
	50^a^	The students irritate the teachers
Subscale III: Students’ Academic Self-Perception	5	Learning strategies that worked for me before continue to work for me now
	10	I am confident about my passing this year
	21	I fell I am being well prepared for my profession
	26	Last year’s work has been a good preparation for this year’s work
	27	I am able to memorise all I need
	31	I have learnt a lot about empathy in my profession
	41	My problem-solving skills are being well developed here
	45	Much of what I have to learn seems relevant to a career in healthcare
Subscale IV: Students’ Perception of Atmosphere	11	The atmosphere is relaxed during ward teaching
	12	This school is well time-tabled
	17^a^	Cheating is a problem in this school
	23	The atmosphere is relaxed during lectures
	30	There are opportunities for me to develop my interpersonal skills
	33	I feel comfortable in class socially
	34	The atmosphere is relaxed during class/seminars/tutorials
	35^a^	I find the experience disappointing
	36	I am able to concentrate well
	42	The enjoyment outweighs the stress of the course
	43	The atmosphere motivates me as a learner
	49	I feel able to ask the questions I want
Subscale V: Students’ Social Self-Perception	3	There is a good support system for students who get stressed
	4^a^	I am too tired to enjoy the course
	14	I am rarely bored in this course
	15	I have good friends in this course
	19	My social life is good
	28	I seldom feel lonely
	46	My accommodation is pleasant

^a^were reverse scored

**Fig. 1:**
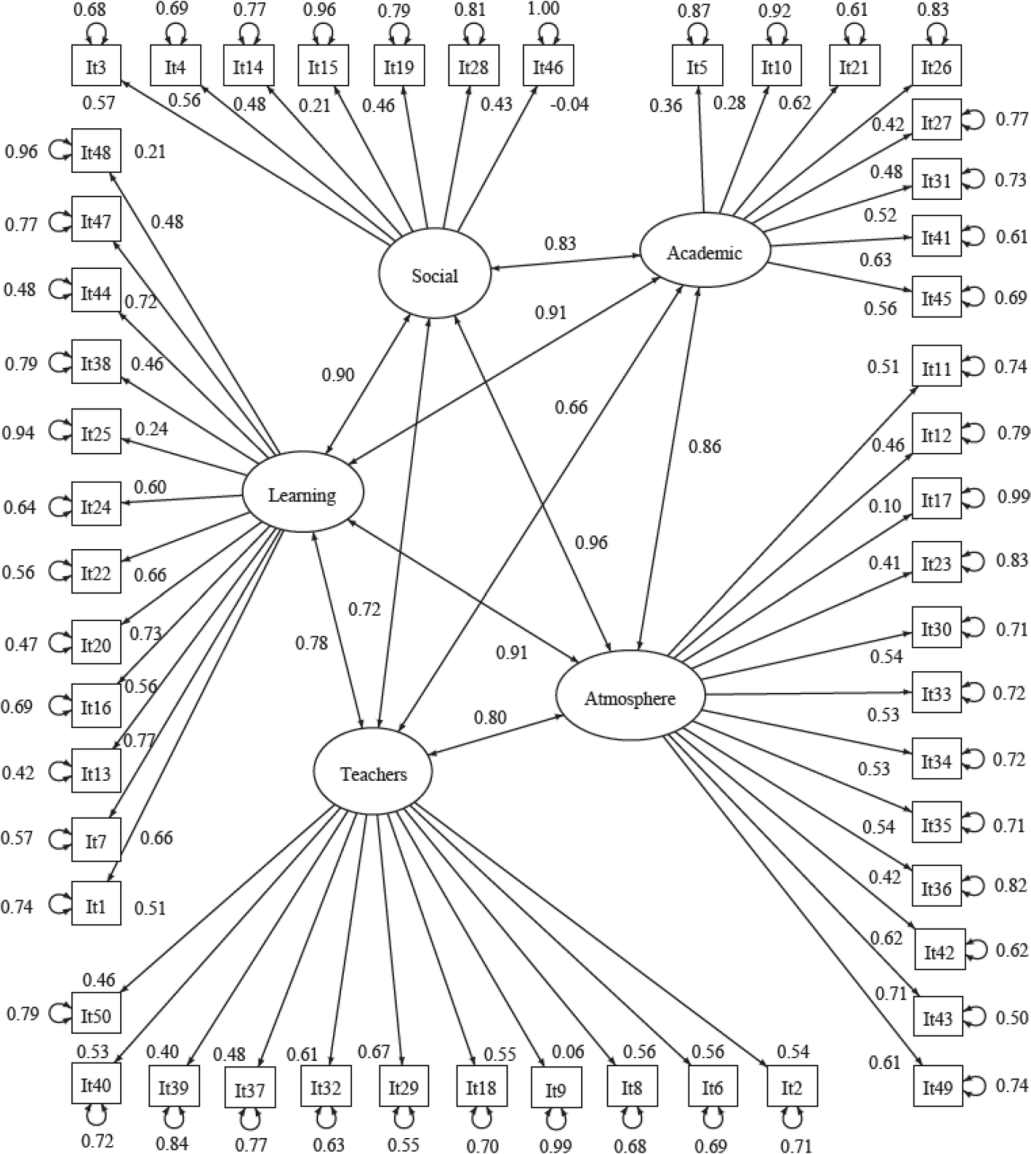
Study group– flow chart

The DREEM questionnaire was distributed among students of all years – from the first to the fifth year of medicine and dentistry at the Medical University of Lublin and the Medical University of Gdańsk. Overall, 650 students participated in the study. The characteristics of the study group are given in Table 2 and Fig. 1. The validated questionnaire in Polish is shown in Table 3. The study was conducted from April to June 2022 and was approved by the Bioethics Committee at the Medical University of Lublin and the Bioethics Committee at the Medical University of Gdańsk, as well as by the authorities of both universities. The deans of the different schools of dentistry gave permission for the study to be carried out and the collaborators involved in the different schools received written instructions on how to implement the project. One of the authors was also conducting the research at both universities.

**Table 2:** Characteristics of the study group *n* = 650

Characteristic	Both universities	Medical University of Lublin	Medical University of Gdańsk	*p* value^a^
Response rates	650/766 (85%)	376/418 (89%)	274/348 (78%)	
Age in years, SD	22.53 ± 2.69	22.21 ± 2.38	22.97 ± 3.0	
Gender				0.596
Male n (%)	152 (23.3%) Missing value = 1	85 (22.6%)	67 (24.5%)	
Female	498 (76.6%)	291 (77.4%)	207 (75.5%)	
Class year n (%)				0.428
Year 1	127 (19.5%)	79 (21%)	48 (17.5%)	
Year 2	136 (20.9%)	71 (18.9%)	65 (23.7%)	
Year 3	124 (19.0%)	75 (19.9%)	49 (17.9%)	
Year 4	133 (20.4%)	73 (19.4%)	60 (21.9%)	
Year 5	130 (19.9%)	78 (20.7%)	52 (19%)	

^a^Pearson’s chi-squared test

**Table 3:** The Polish Dundee Ready Education Environment Measure (DREEM)—items grouped by subscale

Scale	Item	Question
Podskala I: Postrzeganie kształcenia przez studentów	1	Jestem zachęcany(-na) do udziału w zajęciach. percepcja
	7	Nauczanie jest często inspirujące
	13	Nauczanie jest zorientowane na studenta(-kę)
	16	Nauczanie pomaga rozwinąć moje kompetencje
	20	Nauczanie jest dobrze zorganizowane
	22	Nauczanie pomaga rozwinąć moją pewność siebie
	24	Czas przeznaczony na nauczanie jest dobrze wykorzystywany
	25	W nauczaniu zbyt duży nacisk kładzie się na naukę faktów
	38	Znam cele zajęć
	44	Nauczanie zachęca mnie do aktywnego uczenia się
	47	Nacisk położony jest na uczenie się długoterminowe zamiast krótkoterminowego
	48	Nauczanie jest zbyt zorientowane na prowadzącym zajęcia
Podskala II: Postrzeganie nauczycieli przez studentów	2	Nauczyciele są kompetentni
	6	Nauczyciele mają cierpliwość do pacjentów
	8	Nauczyciele ośmieszają swoich studentów
	9	Nauczyciele są autorytarni
	18	Wydaje się, że nauczyciele wykazują skuteczne umiejętności komunikacyjne z pacjentami
	29	Nauczyciele potrafią dobrze przekazywać studentom informacje zwrotne
	32	Nauczyciele przeprowadzają konstruktywną krytykę
	37	Nauczyciele podają zrozumiałe przykłady
	39	Nauczyciele wpadają w złość podczas zajęć
	40	Nauczyciele są dobrze przygotowani do swoich zajęć
	50	Studenci irytują nauczycieli
Podskala III: Akademicka autopercepcja studentów	5	Strategie uczenia się, które wcześniej się sprawdzały w moim przypadku, nadal są skuteczne
	10	Jestem przekonany(-na), że zdam w tym roku
	21	Czuję, że jestem dobrze przygotowywany (-na) do swojego zawodu
	26	Zeszłoroczna nauka stanowiła dobrą podstawę do nauki w tym roku akademickim
	27	Potrafię zapamiętać wszystko, czego potrzebuję
	31	Wiele się nauczyłem(-am) o empatii w moim zawodzie
	41	Moje umiejętności rozwiązywania problemów są dobrze rozwijane na uczelni
	45	Wiele z tego, czego muszę się nauczyć, wydaje się mieć znaczenie dla kariery w opiece zdrowotnej
Podskala IV: Postrzeganie atmosfery przez studentów	11	Podczas nauczania klinicznego panuje swobodna atmosfera
	12	Plan zajęć jest dobrze ułożony
	17	Oszukiwanie (ściąganie) jest problemem na zajęciach
	23	Podczas wykładów panuje swobodna atmosfera
	30	Mam możliwość rozwijania umiejętności interpersonalnych
	33	Czuję się dobrze na zajęciach, pod względem relacji interpersonalnych
	34	Podczas seminariów/ćwiczeń panuje swobodna atmosfera
	35	Uważam, że to doświadczenie jest rozczarowujące
	36	Potrafię się dobrze skoncentrować
	42	Przyjemność przeważa nad stresem związanym ze studiowaniem medycyny
	43	Atmosfera motywuje mnie do nauki
	49	Czuję, że mogę swobodnie zadawać pytania
Podskala V: Społeczna autopercepcja studentów	3	Istnieje dobry system pomocy dla studentów, którzy nie radzą sobie ze stresem
	4	Jestem zbyt zmęczony(-na), aby cieszyć się tymi studiami
	14	Rzadko się nudzę podczas zajęć
	15	Na tych studiach poznałem(-am) dobrych znajomych
	19	Moje życie towarzyskie jest dobre
	28	Rzadko czuję się samotny(-na)
	46	Miejsce, w którym jestem zakwaterowany(-na) jest wygodne

The questionnaire was delivered to the students during their classes. Before beginning the survey, each collaborator briefly explained the study’s objectives and details of the data processing, placing special emphasis on the importance of voluntary participation and the anonymity of the process.. Data on age, gender, and academic year of each participant was collected.

### Participants and criteria for eligibility

The 418 undergraduate full-time students from the end of the first year through to the fifth year of the Medical University of Lublin and the 348 students from the Medical University of Gdańsk present during the classes when both tools—DREEM and. JHLES were administered were invited to participate in this study.

Inclusion criteria were to be a dentistry student and give consent for participation in the study. Exclusion criteria were: previous participation in a pilot study; lack of consent to participate in the study; and failure to complete the questionnaire twice.

Polish medical schools have a five year curriculum: the first two years are preclinical, followed by two years of clinical activity and the last year mostly comprised of hospital activities. The students were informed that after 35 days they would have both the JHLES and the DREEM retested. Students who agreed to take part in the study did not receive any form of financial gratification. The first round of testing lasted approximately 30 minutesand the second round took about 20 min. To compare both the test and retest data, the students were asked to encode the surveys; the survey was pseudoanonymized, and the students also had the opportunity to fully anonymize it by acquiring a number from the number generator. Surveys that were not encoded or had no pairs were excluded from the study.

Sample size selection was based on the generally accepted rule of thumb that there must be at least 5–15 cases per estimated parameter in confirmatory factor analysis (CFA). Assuming a case number of 10 per parameter for a DREEM questionnaire with 50 parameters, the minimum sample size required was estimated to be up to 500 cases [23, 24].

### Validity analysis

Validity was separated into four phases: (1) translation validity, (2) CFA, (3) concurrent validity, and (4) criterion-related validity.

### Translation validity and transcultural adaptation

First of all, the authors agreed to translate and adapt the questionnaire. Later, the author and two other native Polish speakers who are fluent in English translated the content of the questionnaire into Polish. All three versions were compared and the single final version was agreed upon, which is consistent semantically and conceptually with the original. Consensus was developed for each statement in the questionnaire. Minor changes were made so that the scales were adequate for the Polish academic culture. After translating both questionnaires into Polish, they were sent to two native English speakers, who independently translated them back into English. In this way, four questionnaires were obtained, each of them translated backwards – two versions of the DREEM and two versions of the JHLES. Two versions of the reverse translation questionnaires were sent to the original authors to determine the final version of the questionnaires. The final versions were translated into Polish by the author and an additional two people, (e.g.professional translators) and later submitted for consultation and pilot examination by a group of students at the Polish Society of Dentistry Students. The pilot study involved 15 students who were then excluded from subsequent stages of the study. The pilot study aimed to verify whether the answers provided by students were consistent and provided an opportunity to consult with students regarding the language comprehensibility of the questionnaire. Question 17 required adjustment – we added the word ‘ściąganie’ instead ‘oszukiwanie’ for ‘cheating’ because it is used in Polish as ‘cheating’.

After consultations and the pilot study, the final amendments were made to the questionnaires by the Polish authors.

Cultural adaptation to adjust the questionnaire to the Polish academic environment involved adding female grammatical forms (*feminatywy*): female variants of actors and personal characteristics. In Polish, women are distinguished in terms of their titles, functions, positions, professions, nationalities, backgrounds, faiths, convictions, psychological/physical qualities and activities. This class of lexemes has a permanent female grammatical gender that consists of syntactically independent nouns. It does not include adjectives or verbs, in which case gender is an inflectional category. By adding female grammatical forms, we wanted to address both female and male students.

### Statistical analysis

The basic descriptive statistics for the study group and the DREEM results were calculated, divided into the respective medical universities in which the study was conducted, as well as for the gender and year of study of the respondents. Analysis of questionnaire reliability was performedand diagnostic accuracy was examined using correlation analysis and CFA. The participants of the study provided their sociographic data such as age, year of study, gender, and nationality. The calculations were made using IBM SPSS 28, the confirmatory factor analysis was performed using the R package.

The significance level of statistical tests for the analysis was set at α = 0.05. The CFA model was fitted using the DWLS estimator [25] with the NLMINB optimization method [26]. To measure the association between item score and scale (subscale) score, Spearman’s method was applied and Spearman’s rho statistic (ρ) was used to estimate a rank-based measure of association. The *p*-values were computed via the asymptotic *t* approximation.

Goodness-of-fit indices of the CFA were estimated based on polychoric correlation matrices. Interpretation of the goodness-of-fit indices was based on the following cut-off criteria: RMSEA of < 0.05 indicated a “close fit” (e.g., [27, 28]); CFI and TLI of > 0.95 indicated a relatively good model–data fit in general [29]; GFI of ≥ 0.93 and SRMR of ≤ 0.08 indicated an acceptable fit [30]; AGFI of ≥ 0.9, NFI of ≥ 0.9 [31] IFI ≥ 0.9 indicated a good fit [32], RNI ≥ 0.95 [29], PNFI ≥ 0.50 [33], RFI close to 1 indicated a good fit [34].

Analysis was conducted using the R statistical language (Version 4.1.1; R Core Team, 2021) on Windows 10 Pro 64-bit (build 19,044) and the following packages lavaan (version 0.6.12), performance (version 0.10.0) [35], report (version 0.5.1.3) [36], psych (version 2.1.6) [37], semidag for drawing path diagrams [38], and effectsize (version 0.8.2) [33].

### Results – analysis of validity

The study was conducted at the Medical University of Lublin and another medical university in Poland. The overall response rate was 376 / 418 (89%) for the Medical University of Lublin and 274 / 348 (78%) for the Medical University of Gdańsk.

Correlation between the JHLES and another tool with a similar theoretical concept – DREEM – indicated the relevance of the developed tool. Statistical analysis showed that the results of the JHLES and the DREEM correlate significantly with each other (*p* < 0.001). The reported relationship was positive, that is, the higher the JHLES score, the higher the DREEM score. Pearson’s correlation coefficient was 0.797, which indicated a very strong correlation.

### Reliability analysis

Our study obtained very good reliability rates, with Cronbach’s alpha for all scales being > 0.7 [39]. Only sub-scale 5 achieved a lower Cronbach’s alpha, but this was still > 0.5. Cronbach’s alpha reliability indices were either good (sub-scale I) or acceptable (sub-scales II, III and IV). Sub-scale V showed a lower value of 0.596 with weaker reliability. However,the overall reliability was excellent. Table 4 shows Cronbach’s alpha coefficients for the different global scales and subscales.

**Table 4:** Cronbach’s alpha coefficients for the different global scales and subscales (‘observed values’ and ‘expected values’) in the Dundee Ready Education Environment Measure (DREEM)

DREEM	Items	n	CASES	ALPHA
Global scale	50	650	638	0.929
Subscale				
Subscale I		650	648	0.851 (0.832)
Subscale II		650	648	**0.783 (0,798)**
Subscale III		650	644	0.725 (0.716)
Subscale IV		650	646	0.785 (0.761)
Subscale V	2	650	650	0.596 (0.580)
Global scale—gender				
Women	50	497	489	0.930
Men	50	152	148	0.918
Global scale—year	50			
1st year	50	127	121	0.924
2nd year	50	136	135	0.916
3rd year	50	124	121	0.918
4th year	50	133	133	0.926
5th year	50	130	128	0.939
Global scale—faculty				
Medical University of Lublin	50	274	273	0.937
Medical University of Gdańsk	50	376	365	0.933

Items: number of items in the scale or subscale;* n*: number of questionnaires (participants); cases: number of questionnaires without value lost on which the alpha coefficients were calculated. Cronbach’s alpha ‘expected’ values were calculated using the Spearman–Brown formula. The value in bold is an ‘observed’ value, which is inferior to the ‘expected’ value.

The study was conducted using the test–retest method, which is why the intraclass correlation coefficients (ICCs) for reliability were also calculated. Individual items showed medium and good correspondence, items 4, 12, 15, 19, 21, 27 and 43 showed good correspondence according to the latest and more restrictive the ICCs interpretation criteria [40]. Table 5 shows the ICCs for all the DREEM items.

**Table 5:**
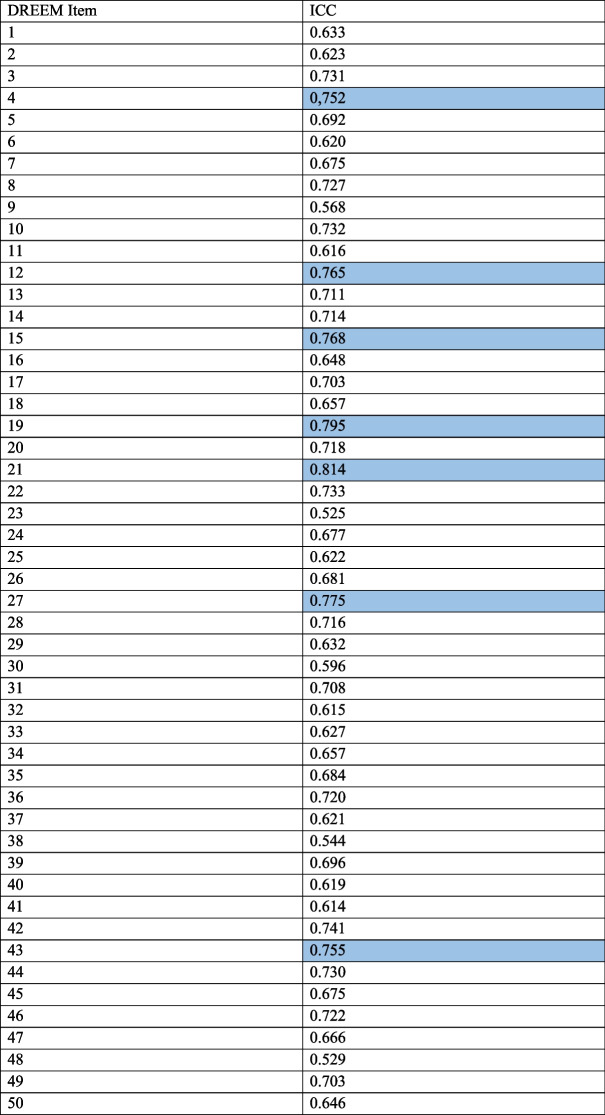
Intraclass correlation coefficients (ICCs) for the Dundee Ready Education Environment Measure (DREEM) items – a two-factor mixed-effects and absolute agreement model

Colour—good correspondence according to the latest and more restrictive the ICCs interpretation criteria

### Confirmatory factor analysis

The number of incomplete questionnaires was low. Of the 17 people (3.0%) who failed to complete all 50 items, the number of missing responses in each item ranged between 1 (*n* = 15, 2.7%) and 2 (*n* = 2, 0.3%). No floor effects and only minor ceiling effects were observed. In Subscale V, one respondent (0.2%) reported the maximum value. Table 6 shows the values of the corrected item–subscale and item–total correlations of the original DREEM structure (50 items). All items were significantly correlated with at least one subscale. Nonsignificant correlation coefficients across the subscales were reported for 4.4% (*n* = 11) of the correlations. Only Item 46 was not significantly correlated with the global questionnaire scale.

**Table 6:**
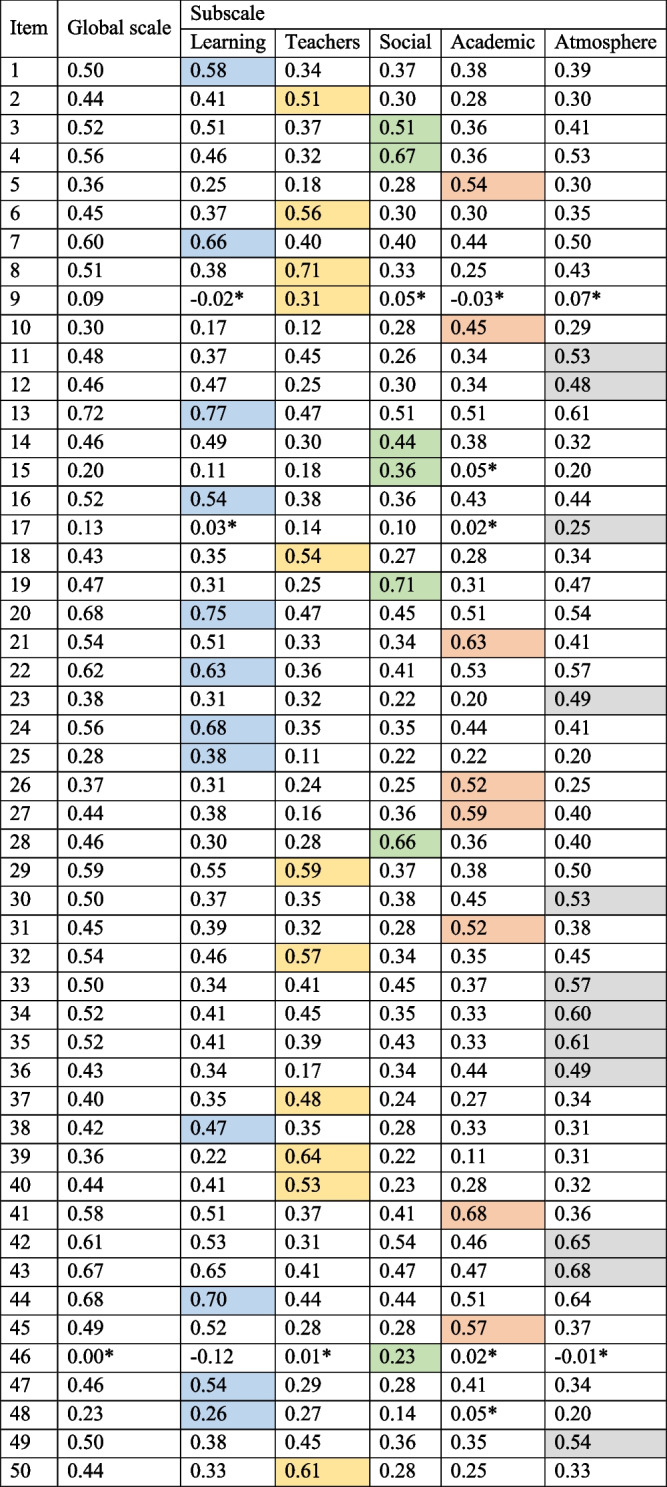
Corrected item-subscale and item-total correlations of the Polish Dundee Ready Education Environment Measure (DREEM) structure (50 items)

^*^correlation significant at the 0.05 levelColour – denotes the original DREEM subscales

**Fig. 2:**
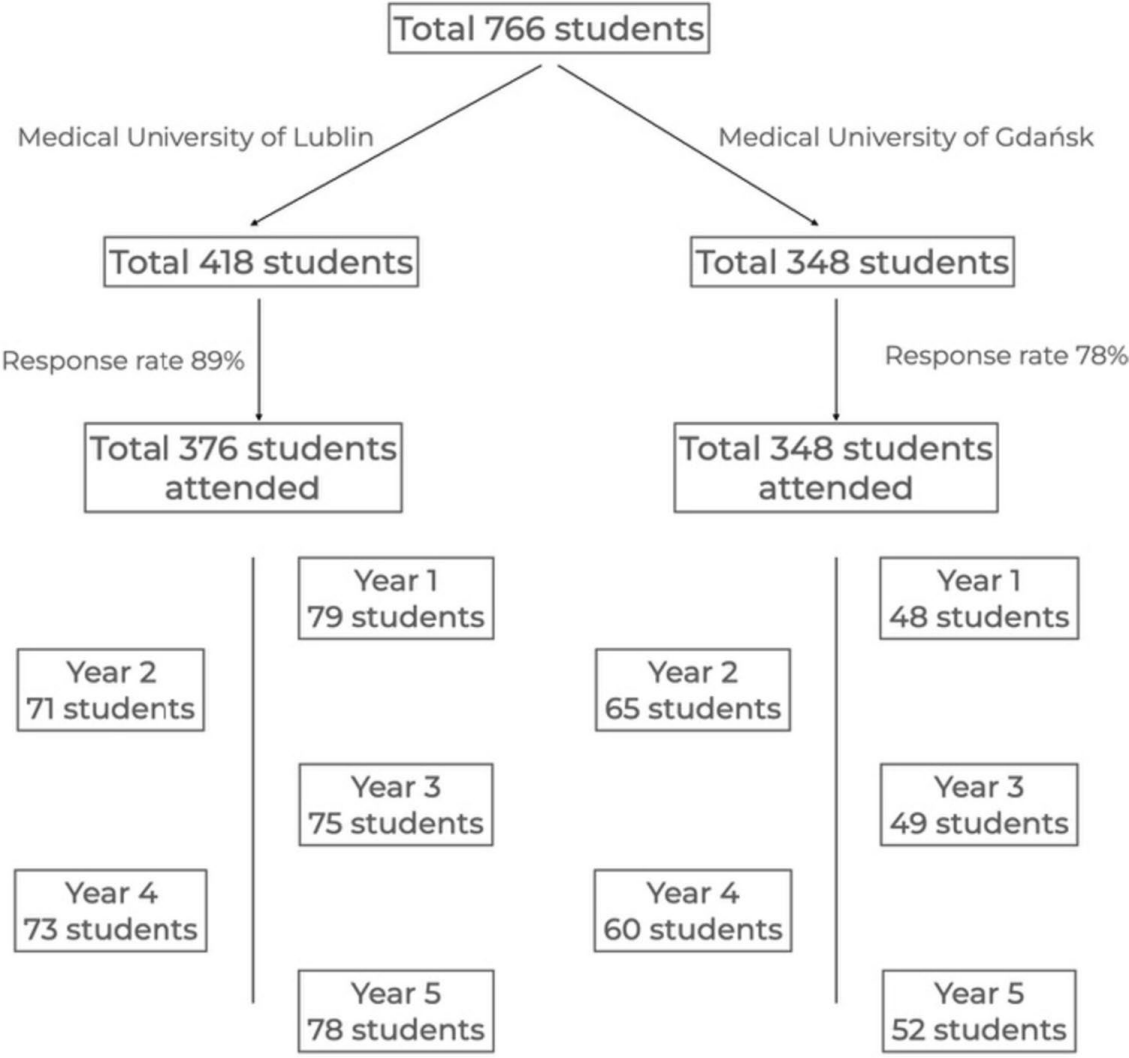
Path diagram of the confirmatory factor model

The model fitting ended normally after 98 iterations. The results of the CFA are shown in a path diagram (Fig. 2). All the items had significant *p*( >|*z*|) estimates, except for Items 9 and 46.

Model tests for the user model (assessing the overall fit and discrepancy between the sample and the fitted covariance matrices) gave: *χ*^*2*^ = 2359.11, *df* = 1165 and *p* < 0.001. Model test for the baseline model gave: χ^2^ = 34,226.83, *df* = 1225 and *p* < 0.001.

The goodness-of-fit indices of the confirmatory factor analysis were as follows: GFI = 0.955, AGFI = 0.951, NFI = 0.931, TLI = 0.962, CFI = 0.964, RNI = 0.964, IFI = 0.964, RFI = 0.928, PNFI = 0.885, SRMR = 0.062, and RMSEA = 0.043 (CI 90% [0.041, 0.046]).

## Discussion

The Polish version of the DREEM questionnaire was adapted in this study. This is the first Polish validation of this questionnaire made on such a large group of subjects, carried out using a less commonly used test–retest method as well as CFA. The validated questionnaire appears to be reliable, as evidenced by its internal consistency and the test-restest consistency, indicateing a sufficient stability of the results over time. Previously Zawislak et al. evaluated the DREEM quesstionnaire via a different methodbut did not confirm the original structure [41].

CFA allowed a model to be obtained with good parameters of goodness to fit. The items correlate positively, and each item correlates with its own subscale. In our study, the DREEM questionnaire was used on the largest study group so far (*n* = 650) and correlation between the average scores for all of the items between both periods was very high (Pearson’s correlation coefficient = 0.790; *p* < 0.001).

### Analysis of the reliability of the DREEM scale

Our study has obtained very good reliability rates, with Cronbach’s alpha ratio > 0.7 for all scales. Only sub-scale V achieved a lower Cronbach alpha score, but still > 0.5. The results by other authors who have carried out the DREEM validation are listed in Table 7 and we obtained similar results to these authors. This may be due to the fact that Cronbach's alpha is affected by the length of the questionnaire, as well as the correlation between items within each scale. We also checked the expected value of Cronbach's alpha with the split-half method for sub-scales, as in the Greek and Spanish validation of the questionnaire [5, 9]. Cronbach’s alpha should be higher than the expected value and only that for the second sub-scale is slightly lower.

**Table 7:** Comparison of internal consistency of validated Dundee Ready Education Environment Measure (DREEM) versions in different countries

Country	Cronbach alfa total scale	Cronbach alfa—subscales
Poland—our	0.929	I 0.851, II 0.783, III 0.725 IV 0.785, V 0.596
Spain	0.92	I 0.75, II 0.79, III 0.69 IV 0.75, V 0.57
Greece	0.9	I 0.79, II 0.78, III 0.69 IV 0.68, V 0.48
Sweden	0.93	I 0.807, II 0.785, III 0.720 IV 0.786, V 0.689
Poland—UJ	0.93	I 0.86, II 0.82, III 0.61 IV 0.75, V 0.61
Indonesia	0.83	III 0.594, V 0.32, the other subscales > 0.7
Pakistan	0,89	I 0.72, II 0.73, III 0.67 IV 0.64, V 0.38
Iran	0,914	I 0.722, II 0.739, III 0.759 IV 0.771, V 0.446
Germany	0.92	I 0.84, II 0.75, III 0.68 IV 0.75, V 0.57
Mexico	0.93	I 0.84, II 0.8, III 0.76 IV 0.78, V 0.56
USA	0.93	I 0.85, II 0.79, III 0.81 IV 0.68, V 0.72
Ireland	0.89	I 0.78, II 0.69, III 0.74 IV 0.56, V 0.55

### Analysis of the validity of the DREEM scale

Recently, Jakobsson et al. in Sweden, Hammond et al. in Ireland, and Yusoff in Malaysia explored the construct validity of the DREEM for medical students [42–44]. Applying a CFA, these authors concluded that the putative five-factor model proposed by the developers of the DREEM is not supported and may be in need of revision. Hammond et al. stated that because their findings were based on Irish medical students, it is unlikely that these weaknesses can be attributed to translation factors [44]. Jakobsson et al. stated that the original model was developed by a qualitative method, which could explain the differences [43]. Some authors questioned the original structure of the DREEM and speculated that it should be a four-factor [45] or even a one-factor [44] scale instrument. The results of some studies indicate that single or even several items should be removed from the original DREEM scale [6, 10]. Jakobsson et al. state that the original model was developed by a qualitative method and this may cause differences in the structure of the tool by other researchers [43].

The Korean validation conducted by Kim et al. using CFA did not allow to confirm the original structure of the tool to be confirmed, the authors obtained unsatisfactory measures of the goodness of fit of the model. Statistical analysis with 451 data sets showed that the RMSEA was 0.06, the GFI was 0.75, and the TLI was 0.73 [11]. The Spanish validation of the DREEM conducted by Tomas et al. confirmed the original structure and the authors obtained satisfactory results (GFI and AGFI 0.9, tSRMR 0.08 and RMSEA < 0.06) [5].

Our study also confirmed the original structure of the DREEM tool Although the result of the chi-square test may suggest that the model does not adequately fit, it should be noted that this test depends greatly on the sample size and that the value of the ‘relative chi-square’ (the chi-square statistic divided by its degrees of freedom) in this case was 2.02. Thus, the relative chi-square was calculated as < 5 and the result can be considered acceptable. In addition, the goodness-of-fit indices of the CFA confirmed the suitability of the model. The GFI values were approximately 0.9, the cut-off point that is traditionally considered acceptable. The SRMR was 0.053 and the RMSEA was < 0.06, both within the cut-off limits proposed by Hu and Bentler for an acceptable fit [29]. Consequently, all indices had acceptable values (close to 1 or 0, depending on the case), and there was consistency in the results, which shows that the DREEM model is supported by the data.

### Limitations

Our study was conducted in two out of ten medical universities in Poland. The results of our study assessing the educational environment using the DREEM questionnaire should therefore be considered carefully when generalizing the results to other institutions. Secondly, both questionnaires – the JHLES and the DREEM—were completed by students on the same day. This may result in a stronger correlation between the instruments than if they were completed at different times.

## Conclusions

Our study provided good evidence for the reliability and validity of the Polish version of the DREEM. In conclusion, the Polish-language version of the DREEM questionnaire is a reliable and valid instrument for analysing the learning environment for dental students, and its factor structure is supported by the data. The use of standardized tools for evaluation of the educational environment will improve the functioning of the Polish educational environment and will also enable its comparison with other countries.

## Data Availability

The datasets generated and/or analysed during the current study are available in Mendeley Data https://doi.org/10.17632/36zpwkbny3.1https://data.mendeley.com/datasets/36zpwkbny3.

## References

[1] Lizzio A, Wilson K, Simons R (2002). University students’ perceptions of the learning environment and academic outcomes: implications for theory and practice. Stud High Educ.

[2] Pimparyon P, Caleer SM, Pemba S. Roff S. Educational environment, student approaches to learning and academic achievement in a Thai nursing school. Med Teach. 2000;22(4):359–3.

[3] Roff S, McAleer S, Harden RM, Al-Qahtani M, Ahmed AU, Deza H (1997). Development and validation of the Dundee Ready Education Environment Measure (DREEM). Med Teach.

[4] Aguilar-Barojas S, Jiménez-Sastré A, Castillo-Orueta ML (2018). Validación de la traducción al idioma español del Dundee Ready Education Environment Measure. Investigación En Educación Médica.

[5] Tomás I, Casares-De-Cal MA, Aneiros A, Abad M, Ceballos L, Gómez-Moreno G (2014). Psychometric validation of the Spanish version of the Dundee ready education environment measure applied to dental students. Eur J Dent Educ.

[6] Koohpayehzadeh J, Hashemi A, Arabshahi KS, Bigdeli S, Moosavi M, Hatami K (2014). Assessing validity and reliability of Dundee ready educational environment measure (DREEM) in Iran. Med J Islam Repub Iran.

[7] Shan T, Wang J (2011). Reliability and validity of a Chinese version of the Dundee ready education environment measure in the postgraduate context. Med Teach.

[8] Rotthoff T, Ostapczuk MS, De Bruin J, Decking U, Schneider M, Ritz-Timme S (2011). Assessing the learning environment of a faculty: psychometric validation of the German version of the Dundee ready education environment measure with students and teachers. Med Teach.

[9] Dimoliatis ID, Vasilaki E, Anastassopoulos P, Ioannidis JPA, Roff S. Validation of the Greek translation of the Dundee ready education environment measure (DREEM). Educ Health. 2010;23(1):348. 20589604

[10] Leman MA (2017). Conctruct validity assessment of Dundee ready educational environment measurement (Dreem) in a school of dentistry. Jurnal Pendidikan Kedokteran Indonesia: The Indonesian Journal of Medical Education.

[11] Kim H, Jeon P, Kim S, Hong J, Kang Y (2021). Cross-Cultural adaptation and validation of the Korean version of the Dundee ready education environment measure (DREEM). Evid Based Complement Alternat Med.

[12] JunaidSarfraz K, Tabasum S, Yousafzai UK, Fatima M (2011). DREEM on: validation of the dundee ready education environment measure in Pakistan.

[13] Al-Naggar RA, Abdulghani M, Osman MT, Al-Kubaisy W, Daher AM, Nor Aripin KNB (2014). The Malaysia DREEM: perceptions of medical students about the learning environment in a medical school in Malaysia. Adv Med Educ Pract.

[14] Prashanth GP, Ismail SK (2018). The dundee ready education environment measure: a prospective comparative study of undergraduate medical students’ and interns’ perceptions in Oman. Sultan Qaboos Univ Med J.

[15] Shochet RB, Colbert-Getz JM, Wright SM (2015). The Johns Hopkins learning environment scale: measuring medical students’ perceptions of the processes supporting professional formation. Acad Med.

[16] Damiano RF, Furtado AO, da Silva BN, Ezequiel O da S, Lucchetti AL, DiLalla LF (2020). Measuring students’ perceptions of the medical school learning environment: translation, transcultural adaptation, and validation of 2 instruments to the Brazilian Portuguese language. J Med Educ Curric Dev.

[17] Zhou Z, Huang R, Zhang G, Gong M, Xian S, Yin H (2022). Nomograms for predicting medical students’ perceptions of the learning environment: multicenter evidence from medical schools in China. Front Public Health.

[18] Tackett S, Bakar HA, Shilkofski NA, Coady N, Rampal K, Wright S (2015). Profiling medical school learning environments in Malaysia: a validation study of the Johns Hopkins Learning Environment Scale. J Educ Eval Health Prof.

[19] Tackett S, Shochet R, Shilkofski NA, Colbert-Getz J, Rampal K, Bakar HA (2015). Learning environment assessments of a single curriculum being taught at two medical schools 10,000 miles apart. BMC Med Educ.

[20] Farajpour A, Esnaashari FF, Hejazi M, Meshkat M (2014). Survey of midwifery students’ perception of the educational environment based on DREEM model at Islamic Azad University of Mashhad in the academic year 2014. Res Dev Med Educ.

[21] Shrestha E, Mehta RS, Mandal G, Chaudhary K, Pradhan N (2019). Perception of the learning environment among the students in a nursing college in Eastern Nepal. BMC Med Educ.

[22] Bakhshialiabad H, Bakhshi G, Hashemi Z, Bakhshi A, Abazari F (2019). Improving students’ learning environment by DREEM: an educational experiment in an Iranian medical sciences university (2011–2016). BMC Med Educ.

[23] Kline RB. Principles and practice of structural equation modeling. New York: Guilford publications; 2023.

[24] Hair JF, Black WC, Babin BJ, Anderson RE (2014). Multivariate data analysis.

[25] Savalei V, Rhemtulla M (2013). The performance of robust test statistics with categorical data. Br J Math Stat Psychol.

[26] Gay DM (1990). Usage summary for selected optimization routines. Comput Sci Tech Rep.

[27] Browne MW. Alternative ways of assessing model fit. Testing Struct Equ Models. 1993;144:136–62.

[28] Jöreskog KG, Sörbom D. LISREL 8: Structural equation modeling with the SIMPLIS command language. Scientific Software international. 1993;122–35.

[29] Hu L, Bentler PM (1999). Cutoff criteria for fit indexes in covariance structure analysis: conventional criteria versus new alternatives. Struct Equ Modeling.

[30] Cho G, Hwang H, Sarstedt M, Ringle CM (2020). Cutoff criteria for overall model fit indexes in generalized structured component analysis. J Mark Anal.

[31] Byrne BM. Structural equation modeling with EQS and EQS/Windows: Basic concepts, applications, and programming. Canada: Sage; 1994.

[32] Bollen KA. Structural equations with latent variables. New York: Wiley; 1989.

[33] Ben-Shachar MDLD, Makowski. effectsize: Estimation of Effect Size Indices and Standardized Parameters. J Open Source Softw. 2020;5:2815.

[34] Lüdecke D, Ben-Shachar M, Patil I, Waggoner P, Makowski D (2021). Performance: an R package for assessment, comparison and testing of statistical models. JOSS.

[35] Lüdecke D, Makowski D, Waggoner P, Patil I. Performance: assessment of regression models performance. R Package Version 0.4. 7. Appl Stat Using R, 440. 2020;5.

[36] Makowski D, Ben-Shachar MS, Patil I, Lüdecke D. Automated results reporting as a practical tool to improve reproducibility and methodological best practices adoption. CRAN Available online at: https://github.com/easystats/report. 2020.

[37] Revelle W. psych: Procedures for Psychological, Psychometric, and Personality Research. Evanston: Northwestern University, Evanston, Illinois R package version 229; 2021.

[38] Mai Y, Xu Z, Zhang Z, Yuan K-H. An Open-source WYSIWYG Web Application for Drawing Path Diagrams of Structural Equation Models. Structural Equation Modeling: A Multidisciplinary Journal. 2023;30(2):328–35.

[39] Taber KS (2018). The use of Cronbach’s alpha when developing and reporting research instruments in science education. Res Sci Educ.

[40] Bobak CA, Barr PJ, O’Malley AJ (2018). Estimation of an inter-rater intra-class correlation coefficient that overcomes common assumption violations in the assessment of health measurement scales. BMC Med Res Methodol.

[41] Zawiślak D, Żur-Wyrozumska K, Habera M, Skrzypiec K, Pac A, Cebula G (2021). Folia Medica CracoviensiaFolia Medica Cracoviensia.

[42] Yusoff MSB (2012). The Dundee ready educational environment measure: a confirmatory factor analysis in a sample of Malaysian medical students. Int J Humanit Soc Sci.

[43] Jakobsson U, Danielsen N, Edgren G (2011). Psychometric evaluation of the Dundee ready educational environment measure: Swedish version. Med Teach.

[44] Hammond SM, O’Rourke M, Kelly M, Bennett D, O’Flynn S (2012). A psychometric appraisal of the DREEM. BMC Med Educ.

[45] Roine I, Molina Y, Cáneo M (2018). A psychometric appraisal of the dundee ready education environment measure in a medical school in Chile. Educ Health.

